# Use of the Workbook Method to estimate the prevalence of chronic hepatitis B infections in the European Union and European Economic Area, 2022

**DOI:** 10.2807/1560-7917.ES.2026.31.14.2500322

**Published:** 2026-04-09

**Authors:** Ana Paula Finatto Canabarro, Erika Duffell, Disa Hansson, Sandra Dudareva, Thomas Seyler, Rene Niehus, Ndeindo Ndeikoundam Ngangro, Ziad El-Khatib, Els Plettinckx, Laure Mortgat, Mariya Tyufekchieva, Fani Theophanous, Vratislav Němeček, Marek Malý, Maria Wessman, Hanna Maria Aavik, Cécile Brouard, Ruth Zimmermann, Dimitrios Paraskevis, Georgia Nikolopoulou, Zsuzsanna Molnár, Emese Kozma, Niamh Murphy, Maria Elena Tosti, Šarlote Konova, Esther Walser-Domjan, Judith Hübschen, Carole Seguin-Devaux, Tanya Melillo, Eline Op de Coul, Tom Woudenberg, Robert Whittaker, Małgorzata Stępień, Magdalena Rosińska, Vítor Cabral Veríssimo, Rui Tato Marinho, Victoria Hernando, Asuncion Diaz, Maria Axelsson, Marie Nordahl, Filippo Pericoli

**Affiliations:** 1 European Centre for Disease Prevention and Control (ECDC), Stockholm, Sweden; 2 Department of Global Public Health, Karolinska Institutet, Stockholm, Sweden; 3 Department of Infectious Disease Epidemiology, Robert Koch Institute, Berlin, Germany; 4 Institute of Public Health, Riga Stradins University, Riga, Latvia; 5 European Union Dugs Agency, Lisbon, Portugal; 6 Institute for Surveillance & Infectious Disease Epidemiology - Austrian Agency for Health and Food Safety (AGES), Vienna, Austria; 7 Department of Epidemiology and Public Health, Sciensano, Brussels, Belgium; 8 Ministry of Health, Bulgaria; 9 Ministry of Health, Nicosia, Cyprus; 10 National Reference Laboratory for Viral Hepatitis, National Institute of Public Health, Prague, Czech Republic; 11 Department of Biostatistics, National Institute of Public Health, Prague, Czech Republic; 12 Department of Infectious Disease Epidemiology and Prevention, Statens Serum Institut, Copenhagen, Denmark; 13 Health Board, Tallinn, Estonia; 14 Santé Publique France, the National Public Health Agency, Saint-Maurice, France; 15 National Public Health Organization, Marousi, Greece; 16 National Center for Public Health and Pharmacy, Budapest, Hungary; 17 HSE Health Protection Surveillance Centre, Dublin, Ireland; 18 National Center for Global Health, Istituto Superiore di Sanità, Rome, Italy; 19 The Centre for Disease Prevention and Control, Riga, Latvia; 20 Office of Public Health, Vaduz, Liechtenstein; 21 Department of Infection and Immunity, Luxembourg Institute of Health, Esch-sur-Alzette, Luxembourg; 22 Infectious Disease Prevention and Control Unit, Health Promotion and Disease Prevention Directorate, Department of Health Regulation, Ministry for Health, Gwardamangia, Malta; 23 Centre for Infectious Disease Control, National Institute for Public Health and the Environment (RIVM), Bilthoven, the Netherlands; 24 Section for Respiratory, Blood-borne and Sexually Transmitted Infections, Department of Infection Control and Vaccines, Norwegian Institute of Public Health, Oslo, Norway; 25 Department of Infectious Disease Epidemiology and Surveillance, National Institute of Public Health NIH – National Research Institute, Warsaw, Poland; 26 Directorate of Information and Analysis, Directorate-General of Health, Lisbon, Portugal; 27 Faculty of Medicine of the University of Lisbon, Lisbon, Portugal; 28 Local Unit of Health Santa Maria, Medical School Lisbon, University of Lisbon, Portugal; 29 National Programme for Viral Hepatitis, Directorate-General of Health, Lisbon, Portugal; 30 National Centre of Epidemiology, Carlos III Health Institute, CIBER in Infectious Diseases (CIBERINFEC), Madrid, Spain; 31 Public Health Agency of Sweden, Solna, Sweden

**Keywords:** Human viral hepatitis, Hepatitis B, Prevalence, Epidemiological method, Epidemiological Monitoring, Europe

## Abstract

**BACKGROUND:**

Up-to-date estimates of chronic hepatitis B virus (HBV) prevalence in both general and key populations are challenging to obtain because of underdiagnosis, heterogeneous surveillance systems and underrepresentation of key populations.

**AIM:**

We aimed to test the Workbook Method to estimate chronic HBV prevalence in 2022 across the EU/EEA, by country and among men who have sex with men (MSM), people who inject drugs (PWID) and migrants.

**METHODS:**

We used the Robert Koch Institute’s version of the Joint United Nations Programme on HIV/AIDS (UNAIDS) Workbook Method to generate HBV prevalence estimates for each EU/EEA country and for MSM, PWID and migrants within each country. We combined data on population size and HBV prevalence for each population group gathered from scientific sources and reviewed by the European Centre for Disease Prevention and Control’s hepatitis national contact points.

**RESULTS:**

Overall, 0.7% (lower bound–upper bound: 0.5–0.9%) of the EU/EEA population (3,226,000 (2,397,000–4,149,000) individuals) were estimated to be living with HBV in 2022. National HBV prevalence ranged from 0.1% (0.1–1.0%) to 3.1% (2.8–3.3%). Prevalence estimates varied from 0.8% (0.5–1.0%) to 10.5% (9.3–11.9%) for migrants, < 0.1% to 8.7% (lower and upper bounds not available) for PWID and from < 0.1% (< 0.1– < 0.1%) to 10.5% (10.2–10.8%) for MSM.

**DISCUSSION:**

Despite limitations, including the inability to address overlapping populations, these estimates confirm substantial chronic HBV prevalence in the EU/EEA, with considerable variation between countries and population groups. This relatively straightforward method offers an alternative means of generating HBV prevalence estimates.

**Key public health message**

**What did you want to address in this study and why?**

We wanted to test the use of a simple and transparent method of estimating how many people are living with chronic hepatitis B (HBV) in European Union/ European Economic Area (EU/EEA) countries, including populations that are often underrepresented in routine data. Reliable estimates are essential for planning effective hepatitis B prevention and care but are difficult to obtain without strong information systems or complex modelling.

**What have we learnt from this study?**

We learnt that the Workbook Method can be adapted to countries with different epidemiological situations and population profiles. However, it has important limitations and depends heavily on data quality. Our results show that an estimated 2,397,000 to 4,149,000 people in the EU/EEA are living with chronic HBV infection, with wide variation between countries and population groups.

**What are the implications of your findings for public health?**

Improved prevalence estimates help countries understand their local epidemics in terms of where HBV is most common and which populations are most affected, supporting more targeted testing, vaccination and treatment efforts. The Workbook Method can be adapted to settings with varying data availability and does not require advanced statistical software or expertise, making it especially useful in countries with weaker information systems.

## Introduction

The latest World Health Organization (WHO) estimates from 2022 suggest that 254 million persons worldwide are living with chronic hepatitis B virus (HBV) infection [1]. Chronic HBV infection can lead to cirrhosis and hepatocellular carcinoma, which are associated with high morbidity and mortality. In 2022, around 1.1 million persons worldwide were estimated to have died from HBV infection [1].

While the widespread implementation of HBV vaccination has reduced HBV transmission across the European Union (EU) and European Economic Area (EEA), chronic infection prevalence remains high in some EU/EEA countries. Previously reported HBV prevalence estimates from studies conducted among the general population reached up to 4.5% [2]. Mathematical modelling indicates that the disease burden associated with chronic HBV infection in Europe will continue to grow unless prevention and control efforts are scaled up [3].

The latest HBV guidelines recommend simplified and expanded treatment for persons with chronic HBV infection, which will result in more persons being eligible for treatment [4]. Implementing these recommendations requires careful planning of services to ensure they are tailored to the needs of the local population, which in turn requires robust, up-to-date prevalence estimates. However, accurately estimating the number of persons living with chronic HBV infection is challenging. First, while routine diagnostics and notification data can serve as a proxy for prevalence for some infections, this is not the case for chronic HBV. Most persons are asymptomatic and notification data are strongly affected by local testing initiatives and surveillance systems. Estimates suggest that only 15.7% of persons living with chronic HBV infection in the WHO European Region have been diagnosed (this estimate is likely to be higher for the EU/EEA) [1]. Additionally, surveillance varies across countries, from mandatory and comprehensive surveillance systems to sentinel and voluntary reporting, with some systems collecting data on persons newly diagnosed with acute or chronic HBV infections, while others only collect data on acute infections [5,6].

Second, in most European countries, certain key populations—such as migrants from countries with a higher HBV endemicity and lower vaccination coverage than the destination country, men who have sex with men (MSM) and people who inject drugs (PWID)—are disproportionately affected by chronic HBV infection, with higher prevalence rates than the general population [2,7]. These population groups often experience structural, social and health-system barriers to access prevention, testing and care, and are often underrepresented in prevalence studies conducted among the general population. Consequently, population-based studies described as ‘representative’ may underestimate the national prevalence of HBV infection.

Given these challenges, researchers have explored indirect methods, such as complex mathematical models, to estimate chronic HBV prevalence in the general population. The Workbook Method is a straightforward alternative developed to estimate infectious disease prevalence in countries with low-level and concentrated epidemics. Once established, this method can be easily reproduced by countries provided that reliable data are available. Originally designed by the Joint United Nations Programme on HIV/AIDS (UNAIDS) to estimate HIV prevalence [8], the Workbook Method has been adapted for other infectious diseases, including HBV and hepatitis C [9,10]. While this method has been applied in Germany and the Netherlands, it has not yet been used to estimate chronic HBV prevalence in other EU/EEA countries.

We aimed to test the use of the Workbook Method to estimate the national chronic HBV infection prevalence in all EU/EEA countries and to determine which key populations had the greatest influence on the overall prevalence estimates.

## Methods

### The Workbook Method

Our study used a modified version of the Workbook Method adapted by the German national public health institute (Robert Koch Institute, RKI) [10] from the original developed by UNAIDS [8]. The RKI-adapted version uses Microsoft Excel worksheets that specify the data needed for each country including chronic HBV prevalence estimates and population size for various key populations disaggregated by age group. Unlike the original UNAIDS workbook, the RKI-adapted version does not require imputation of data such as risk levels for different populations, subnational geographic information or prevalence trends over time. Its outcomes are limited to chronic HBV prevalence estimates at national level and by population group.

We developed a workbook for each EU country (Austria, Belgium, Bulgaria, Croatia, Cyprus, Czechia, Denmark, Estonia, Finland, France, Germany, Greece, Hungary, Ireland, Italy, Latvia, Lithuania, Luxembourg, Malta, the Netherlands, Poland, Portugal, Romania, Slovakia, Slovenia, Spain and Sweden) and three EEA countries (Iceland, Liechtenstein and Norway) using Microsoft Excel 2016.

We calculated the number of persons living with chronic HBV infection in January 2022. If recent data were lacking, estimates were calculated for 2020 or 2021, depending on the latest available data.

### Data sources

We collected information on population size and chronic HBV prevalence for four population groups: migrants, PWID, MSM and the non-key population. Wherever available, lower and upper bound values were collected from data sources and used in the workbook as part of the uncertainty calculations. Otherwise, only point estimates were used.

By default, migrants were defined as individuals born outside the country of study and were stratified by age group (< 5 years old, ≥ 5 years old) and country of birth. The cut-off age of 5 years was applied because the default data source for HBV prevalence (Polaris Observatory [11]) does not provide estimates stratified by vaccination status; although a classification based on vaccination status would have been preferable, this was not feasible given the available data. Migrant population size data were retrieved from Eurostat [12], while chronic HBV prevalence estimates for both host countries and countries of origin were primarily obtained from the Polaris Observatory [11], with alternative sources used where more appropriate. The Polaris Observatory was chosen as the primary source of estimates because of its comprehensive coverage and because it is a data source with which countries in the region are most familiar. The model used by the Polaris Observatory is a dynamic HBV transmission and disease burden Markov model that uses a Delphi process to include data gathered from literature reviews and from interviews with country experts.

Estimates of the PWID population size were retrieved from Thomadakis et al. [13], with HBV prevalence sourced from the European Union Drugs Agency’s Viral hepatitis elimination barometer among people who inject drugs in Europe [14]. By default, MSM population size estimates were based on Marcus et al. [15], with HBV prevalence obtained from Trickey et al. [16]. The non-key population size was derived by excluding migrants, PWID and MSM from the national population and stratified by age group (< 5 years old, ≥ 5 years old). National HBV prevalence for this group was obtained from the Polaris Observatory [11], Trickey et al. [16] or directly from the European Centre for Disease Prevention and Control’s (ECDC) hepatitis national contact points (NCPs) for each country.

Data on estimates of population size and prevalence for each population group included in the country-specific workbooks were shared with the ECDC hepatitis NCPs and other national experts designated by the NCPs. When data were not available, estimates from countries with similar epidemiological characteristics were used for imputation; if this was not possible, the data were considered missing and no further calculations were conducted. The data in the workbooks were reviewed and discussed through an iterative review process that aimed at incorporating the most robust, representative and relevant data available. European Centre for Disease Prevention and Control and national experts deliberated on alternative data options to reach consensus.

The estimated number of persons living with chronic HBV infection in each population group was determined by multiplying the chronic HBV prevalence for the group by the respective population size. For the migrant population, the number of persons living with chronic HBV infection was calculated separately for each country of birth and age group and then aggregated to obtain the total number of migrants living with chronic HBV infection.

To address uncertainties, the lower bound of the population size was multiplied by the lower bound of chronic HBV prevalence. The same applied for the upper bounds.

The estimated number of persons living with chronic HBV infection was presented in absolute values and rounded to the nearest thousand if the count exceeded 1,000 or to the nearest hundred if the count was below 1,000. Chronic HBV prevalence estimates were presented as relative values in the form of percentages, each rounded to a single decimal place.

All estimates presented in the tables are based on the default workbook and country-specific adaptations.

### Use of Bayes’ theorem

In addition, we used Bayes’ theorem to calculate the probability of belonging to a specific population (e.g. MSM) among those living with chronic HBV infection. This probability can reveal which population groups contribute the most to chronic HBV cases and have the greatest influence on the final overall estimates. Consequently, this informs how the final prevalence estimate relies on precise data for a given population.

If *A* denotes the population group and *B* denotes that a person is living with chronic HBV infection, the reverse probability was calculated using the formula:

P(A|B) = [P(B|A) ∙ P(A)]/P(B)

where P(A) is the probability that a person belongs to a population group (e.g. MSM), P(B) is the probability that a person is living with HBV, P(A|B) is the probability that a person belongs to a population group (e.g. MSM) given the person is living with chronic HBV infection, and P(B|A) is the probability that a person is living with chronic HBV infection given the person belongs to a population group (e.g. MSM).

### Sensitivity analysis

From RKI’s workbook [10], we developed a default workbook with standardised data sources to keep a consistent methodological approach across countries. We prioritised approaches that minimised the risk of bias and selected the data source with the widest availability across the studied countries. However, due to country-specific factors–such as data completeness, the dynamics of HBV epidemics and demographic aspects, particularly migration patterns–some NCPs suggested flexibility in the assumptions and tailoring of their country’s workbook. Sensitivity analyses that could be performed due to the availability of relevant data were undertaken to examine the effects of these adaptations and the impact of some key assumptions on the final estimates.

The default data sources and the adaptations applied to each country’s workbook are described in Supplementary Table S1, while the assumptions that shaped the design of the default workbook template and the selection of data sources are detailed in Supplementary Table S2.

#### Potential impact of increased number of Ukrainian refugees (analysed country: Denmark)

The war in Ukraine resulted in a large number of Ukrainian refugees arriving in EU/EEA countries from March 2022 onwards [17]. Since this workbook utilises migrant data from 1 January 2022, the impact of the increased number of Ukrainian migrants in EU/EEA countries was not reflected in the default workbook estimates. The Danish NCP provided complete migrant data for the last quarter of 2023, enabling us to substitute the default migrant data (from the first quarter of 2022) and explore whether the arrival of Ukrainian migrants to Denmark could influence the country’s final estimates of chronic HBV prevalence.

#### Effect of classifying overseas territories as part of their dependent country compared with as ‘unknown’ (analysed country: Malta)

Only sovereign countries were listed as country of birth for migrants in our workbook. Therefore, migrants who were born in an overseas territory were categorised in the workbook as having an ‘unknown’ country of birth and assigned the HBV prevalence of the country they migrated to (e.g. Malta in the Maltese workbook). The Maltese NCP provided information on how many migrants were born in non-sovereign overseas territories. We included these migrants under their respective dependent countries to assess whether this approach could impact Malta's final estimates.

#### Effect of classifying migrants based on their nationality compared to country of birth (analysed country: Portugal)

In the default workbook, migrants were classified based on their country of birth, which informed the migrant's respective chronic HBV prevalence. We assumed that country of birth was a more accurate indicator of chronic HBV prevalence among migrants than nationality. This is because most chronic HBV infections occur in early childhood, so prevalence is more closely related to the country where someone was born, rather than the country they migrated to later in life and where nationality was obtained. However, to assess how our assumption may have influenced the results, we used an additional dataset provided by the Portuguese NCP, which classifies migrants by their registered nationality rather than country of birth. This data was incorporated into the Portuguese workbook to investigate how different definitions of migrants' origin could impact the final estimates for this country.

#### Effect of using chronic hepatitis B virus prevalence estimates for migrant’s country of birth from different data sources (analysed country: Germany)

Most chronic HBV prevalence estimates for migrants’ countries of birth were retrieved from the Polaris Observatory [11]. However, based on the suggestion of the German NCP, we also considered another data source that compiled estimates using a different approach [18]. We used this new information to test how the choice of data source for migrants’ chronic HBV prevalence could affect the final estimates for Germany.

#### Effect of using an age cut-off of 25 years compared with 5 years (analysed country: Poland)

Hepatitis B virus infections have considerably decreased in the age groups of vaccinated cohorts since the WHO published its recommendation for vaccinating infants against HBV in 1992 [19]. In Poland, universal HBV vaccination of newborns has been continuously implemented since 1996 [20]. Consequently, the cohort of newborns vaccinated against HBV approximately corresponds to the age group of 0–24 years by 2022. Based on this, the Polish NCP adapted the Workbook Method using 25 years as the cut-off age for their non-key population. The results were then compared with the cut-off of 5 years old used in the default workbook.

### Results review by country

All countries were invited to review the final estimates and provide comments based on national surveillance data and expert knowledge, where relevant. The comments provided by each country are presented in Supplementary Table S3.

## Results

We calculated national estimates of chronic HBV infection prevalence for all 30 countries in the EU/EEA. For 21 countries, we estimated prevalence for 2022. We generated 2021 estimates for Croatia, Greece, Malta, Poland, Portugal and Sweden and 2020 estimates for Estonia, France and Germany owing to the year of the most recent and best quality data available for each country.

At the EU/EEA level, the estimated chronic HBV prevalence was 0.7% (lower bound–upper bound (LB–UB): 0.5–0.9%), with a total of 3,226,000 (LB–UB: 2,397,000–4,149,000) persons living with chronic HBV infection (Table). Two thirds (66%) of those living with chronic HBV belonged to the non-key population, followed by the migrant population, which accounted for 30% of persons living with a chronic HBV infection.

**Table:** Estimated chronic hepatitis B virus prevalence, with lower and upper bounds, and estimated number of persons living with chronic hepatitis B virus infection at national level and within different population groups, European Union/ European Economic Area countries and the European Union/ European Economic Area region^a^, 2020–2022

Country (estimated year)^b^	National level				Migrants						PWID				MSM			
	Prevalence (%)	LB–UB (%)	n	LB–UB	Prevalence %		LB–UB (%)	n		LB–UB	Prevalence %	LB–UB (%)	n	LB–UB	Prevalence %	LB–UB (%)	n	LB–UB
Austria (2022)	0.9	0.6–1.0	77,000	54,000–86,000	1.8	1.3–2.2		32,000	24,000–41,000		3.1	3.1–3.1	1,000	1,000–1,000	1.6	1.5–1.7	1,000	1,000–1,000
Belgium (2022)	0.7	0.6–0.9	86,000	68,000–104,000	1.8	1.4–2.2		37,000	29,000–47,000		1.9	1.9–1.9	400	400–400	0.8	0.8–0.8	1,000	1,000–1,000
Bulgaria (2022)	2.6	1.8–4.2	181,000	120,000–286,000	1.8	1.2–2.2		4,000	3,000–5,000		8.6	8.6–8.6	6,000	5,000–6,000	7.9	7.6–8.2	1,000	1,000–1,000
Croatia (2021)	0.6	0.5–1.0	24,000	20,000–40,000	1.6	1.2–2.0		500	300–600		0.8	0.3–1.9	100	< 100–300	2.8	2.7–2.8	700	600–700
Cyprus (2022)	2.7	2.4–3.0	24,000	22,000–28,000	10.5	9.3–11.9		22,000	19,000–24,000		4.2	4.2–4.2	100	< 100–100	< 0.1	< 0.1– < 0.10.0–0.0	< 100	< 100– < 100
Czechia (2022)	0.5	0.3–0.6	50,000	28,000–62,000	1.8	1.5–2.4		8,000	7,000–11,000		1.2	1.2–1.2	1,000	1,000–1,000	0.9	0.9–1.0	400	400–400
Denmark (2022)	0.5	0.4–0.7	29,000	24,000–39,000	2.1	1.6–2.8		18,000	13,000–23,000		1.3	1.2–1.3	600	600–700	0.6	0.6–0.6	600^c^	600–600^c^
Estonia (2020)	0.8	0.6–1.0	11,000	9,000–13,000	1.4	0.8–1.6		3,000	2,000–3,000		8.0	8.0–8.0	3,000	2,000–3,000	3.6	3.5–3.7	300	200–400
Finland (2022)	0.3	0.2–0.4	19,000	12,000–21,000	1.8	1.3–2.2		8,000	6,000–10,000		0.6	0.5–0.7	300	200–400	0.8	0.7–0.9	800^c^	600–900^c^
France (2020)	0.4	0.3–0.6	273,000	199,000–389,000	2.2	1.7–2.8		186,000	146,000–232,000		0.7	0.3–1.5	2,000	900–5,000	1.0	0.9–1.0	9,000^c^	9,000–9,000^c^
Germany (2020)	0.3	0.2–0.5	282,000	187,000–431,000	2.1	1.5–2.7		164,000	119,000–208,000		1.1	1.1–1.1	5,000	5,000–5,000	1.0	1.0–1.0	6,000	6,000–7,000
Greece (2021)	1.9	1.6–2.2	191,000	162,000–224,000	4.2	3.0–6.2		32,000	23,000–48,000		2.1	2.1–2.1	400	400–400	2.0	2.0–2.0	2,000	2,000–2,000
Hungary (2022)	0.5	0.5–0.6	51,000	49,000–62,000	1.9	1.7–2.3		12,000	10,000–14,000		2.2	2.2–2.2	500	500–600	5.7	5.5–5.8	3,000	3,000–3,000
Iceland (2022)	0.3	0.2–0.5	1,000	900–2,000	1.4	1.0–1.8		1,000	700–1,000		< 0.1	< 0.1– < 0.1	< 100	< 100– < 100	Missing information			
Ireland (2022)	0.4	0.3–0.5	19,000	14,000–24,000	1.5	1.2–2.0		17,000	13,000–21,000		0.8	0.8–0.9	100	100–200	0.3	0.2–0.4	300	200–400
Italy (2022)	0.7	0.5–0.9	434,000	293,000–535,000	2.5	1.9–3.3		156,000	120,000–204,000		2.6	2.6–2.6	6,000	6,000–6,000	3.3	3.2–3.4	11,000	11,000–12,000
Latvia (2022)	1.0	0.9–1.1	20,000	17,000–21,000	1.8	1.0–2.2		4,000	2,000–5,000		3.6	3.6–3.6	1,000	900–1,000	3.9	3.8–4.0	400	400–400
Liechtenstein (2022)	0.6	0.3–0.7	200	100–300	0.8	0.5–1.0		200	100–300		0.2	0.2–0.2	< 100	< 100– < 100	0.2	0.2–0.3	< 100	< 100– < 100
Lithuania (2022)	2.1	1.0–2.5	60,000	28,000–71,000	1.8	1.2–2.3		3,000	2,000–4,000		4.9	4.9–4.9	2,000	1,000–2,000	5.8	5.6–5.9	800	800–800
Luxembourg (2022)	0.9	0.7–1.1	6,000	5,000–7,000	1.6	1.2–1.9		5,000	4,000–6,000		1.5	1.5–1.5	< 100	< 100– < 100	< 0.1	< 0.1– < 0.1	< 100^c^	< 100– < 100^c^
Malta (2021)	0.9	0.7–1.2	5,000	4,000–6,000	2.0	1.4–2.7		3,000	2,000–4,000		< 0.1	< 0.1– < 0.1	< 100	< 100– < 100	5.9	5.7–6.1	300	300–300
The Netherlands (2022)	0.4	0.3–0.5	65,000	46,000–85,000	2.2	1.7–2.7		55,000	43,000–69,000		3.0	1.0–6.0	300	< 100–800	0.6	0.6–0.6	1,000	1,000–1,000
Norway (2022)	0.4	0.3–0.5	24,000	19,000–30,000	2.1	1.5–2.7		19,000	14,000–25,000		0.8	0.4–1.0	200	100–300	0.7	0.7–0.7	400^c^	400–400^c^
Poland (2021)	0.7	0.5–1.0	284,000	201,000–366,000	0.9	0.7–1.2		7,000	5,000–9,000		2.9	2.9–2.9	600	500–700	1.0	1.0–1.0	600^c^	600–700^c^
Portugal (2021)	1.4	1.0–1.7	142,000	107,000–174,000	3.4	2.7–3.7		37,000	30,000–41,000		6.0	6.0–6.0	3,000	3,000–3,000	2.6	2.5–2.7	3,000	2,000–3,000
Romania (2022)	3.1	2.8–3.3	585,000	527,000–624,000	2.5	2.0–2.9		8,000	7,000–10,000		8.7	8.7–8.7	24,000	23,000–24,000	10.5	10.2–10.8	300^c^	300–300^c^
Slovakia (2022)	0.1	0.1–1.0	5,000	3,000–51,000	0.8	0.6–1.0		2,000	1,000–2,000		1.9	1.9–1.9	1,000	1,000–1,000	1.1	1.1–1.2	200	200–200
Slovenia (2022)	0.7	0.6–0.8	15,000	12,000–16,000	1.8	1.3–2.2		5,000	4,000–6,000		1.4	1.4–1.4	200	100–200	1.9	1.8–1.9	700^c^	700–700^c^
Spain (2022)	0.4	0.2–0.5	171,000	104,000–254,000	1.1	0.8–1.6		82,000	61,000–119,000		5.6	5.4–5.6	2,000	2,000–2,000	1.2	1.2–1.2	4,000	4,000–4,000
Sweden (2021)	0.5	0.4–0.6	55,000	42,000–65,000	2.3	1.6–2.8		46,000	33,000–57,000		2.1	2.1–2.1	500	500–500	0.7	0.6–0.7	500	500–500
EU/EEA region (2022)	0.7	0.5–0.9	3,226,000	2,397,000–4,149,000	2.0	1.5–2.6		977,000	740,000–1,246,000		3.0	2.9–3.1	61,000	57,000–66,000	1.4	1.3–1.4	49,000	47,000–50,000

LB: lower bound; MSM: men who have sex with men; PWID: people who inject drugs; UB: upper bound.

^a^ Chronic hepatitis B virus (HBV) prevalence estimates were rounded to a single decimal place while the number of persons living with chronic HBV infection to the nearest thousand if the count exceeded 1,000, and to the nearest hundred if the count was below 1,000.

^b^ Based on the year of the population size estimate.

^c^ The MSM population size estimate was considered to be a ‘questionable or unreliable value’ in the original study [15].

Chronic HBV infection prevalence estimates varied between countries and population groups (Table). National point estimates ranged from 0.1% in Slovakia to 3.1% in Romania. The estimated number of persons living with chronic HBV infection ranged from 200 in Liechtenstein to 585,000 in Romania. Among migrant populations, the estimated point prevalence varied from 0.8% in Liechtenstein and Slovakia to 10.5% in Cyprus, with the estimated number of migrants living with chronic HBV infection ranging from 200 in Liechtenstein to 186,000 in France. For PWID, the estimated prevalence ranged from 0.0% in Malta and Iceland to 8.7% in Romania. Similarly, the estimated prevalence among MSM varied from 0.0% in Cyprus and Luxembourg to 10.5% in Romania.

In total, 29 of 30 countries reviewed chronic HBV estimates for their country. Of these, 15 of 29 provided comments, with 8 of 15 stating that the HBV workbook estimates were higher than expected and/or presented in the literature.

### Use of Bayes’ theorem

The Bayes’ theorem calculations showed that the distribution of persons living with chronic HBV infection across the different population groups was heterogenous. For the majority of countries (16 of 30), over 50% of the estimated persons living with chronic HBV infection were among non-key populations, ranging from 53% in Finland to 97% in Poland. However, for 12 of 30 countries, most estimated persons living with chronic HBV infection were migrant populations, ranging from 55% in Malta to 97% in Liechtenstein. Spain presented an almost equal representation of persons living with chronic HBV infection among migrants (49%) and non-key populations (48%). In Estonia, more than 20% of persons living with chronic HBV infection were estimated to be PWID, and in Malta, over 6% were estimated to be among MSM (Figure).

**Figure:**
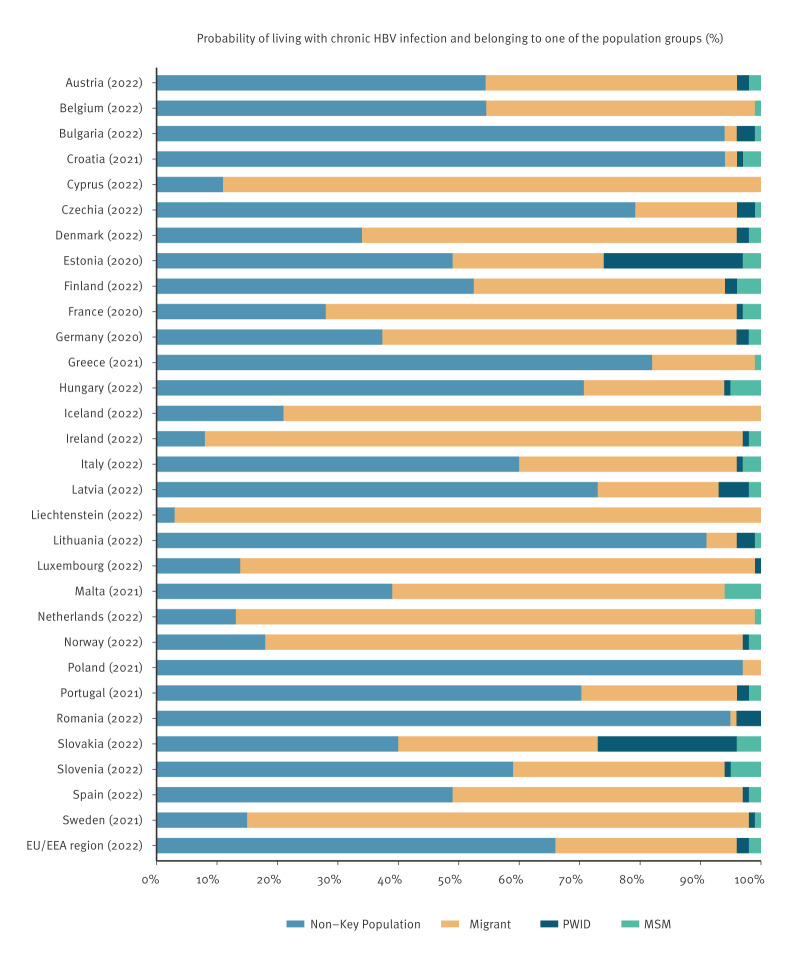
Probability of belonging to one of the studied population groups (migrants, people who inject drugs, men who have sex with men) among those living with chronic hepatitis B infection, European Union/ European Economic Area countries and the European Union/ European Economic Area region, 2020–2022

EEA: European Economic Area; EU: European Union; MSM: men who have sex with men; PWID: people who inject drugs.

### Sensitivity analysis

#### Potential impact of increased number of Ukrainian refugees (analysed country: Denmark)

Comparing the Danish population sizes for the first quarter of 2022 with those for 2023 [21], the general population increased by 1% (2022: 5,873,420; 2023: 5,959,464), the migrant population by 11% (2022: 847,041; 2023: 938,693) and the migrant population from Ukraine by 170% (2022: 16,422; 2023: 44,286). While the absolute number of persons living with chronic HBV infection over this period changed, the overall national chronic HBV prevalence remained consistent at 0.5% (LB–UB: 0.4–0.7%) for both years. Similarly, the chronic HBV estimate among migrants was unchanged at 2.1%, although the LB and UB slightly narrowed in 2023 (1.6–2.7% vs 1.6–2.8% in 2022). Due to the increased proportion of migrants in the Danish population in 2023, the probability of being a migrant among those living with chronic HBV infection rose modestly from 62.1% in 2021 to 64.7% in 2023.

#### Effect of classifying overseas territories as part of their dependent country compared with as ‘unknown’ (analysed country: Malta)

In total, 170 migrants (0.1% of the total migrant population in Malta) were reclassified based on their country of birth. Neither the national chronic HBV prevalence (0.9%; LB–UB: 0.7–1.2%) nor the migrant chronic HBV prevalence (2.0%; LB–UB: 1.4–2.7%) were affected by the reclassification.

#### Effect of classifying migrants based on their nationality compared to country of birth (analysed country: Portugal)

With the reclassification from country of birth to nationality, the number of migrants in Portugal dropped from 1,089,000 to 736,000, respectively, (reduction of 32%) as several individuals who were born abroad had Portuguese citizenship. The change was greater among Lusophone countries, such as Angola (from 156,998 to 29,758, respectively) and Mozambique (from 65,270 to 4,480, respectively), and also for France (from 103,285 to 27,085, respectively). The national chronic HBV prevalence changed from 1.4% (LB–UB: 1.0–1.7%) to 1.2% (LB–UB: 0.9–1.5%) with the alternative migrant classification. Migrant chronic HBV prevalence also decreased from 3.4% (LB–UB: 2.7 –3.7%) to 2.8% (LB–UB: 2.3–3.3%), as well as the probability of being a migrant among those living with chronic HBV infection (from 25.8% to 13.6%).

#### Effect of using chronic hepatitis B virus prevalence estimates for migrant’s country of birth from different data sources (analysed country: Germany)

Comparing the two data sources, chronic HBV prevalence among migrants varied in direction and magnitude depending on the country of birth. The new approach increased the national chronic HBV prevalence from 0.3% (LB–UB: 0.2–0.5%) to 0.4% (LB–UB: 0.3–0.6%) and the prevalence in the migrant population from 2.1% (LB–UB: 1.5–2.7%) to 2.7% (LB–UB: 2.5–3.0%). It also affected the Bayes’ theorem results, leading to a six-percentage point increase in the probability of being a migrant among those living with chronic HBV infection (from 58% to 64%).

#### Effect of using an age cut-off of 25 years compared with 5 years (analysed country: Poland)

Changing the cut-off age from 5 to 25 years decreased the Polish national chronic HBV estimate from 0.7% (LB–UB: 0.5–1.0%) to 0.6% (LB–UB: 0.5–0.9%), respectively, the estimated number of persons living with chronic HBV infection from 284,000 to 243,000, respectively, (reduction of 14%) and the probability that a person living with chronic HBV infection did not belong to any studied key population by 0.5% (from 97% to 96.5%, respectively).

## Discussion

This study tested the use of the Workbook Method to estimate chronic HBV prevalence for all EU/EEA countries and identified which population groups have the greatest impact on national estimates. Most national estimates were provided for 2022, although some countries’ estimates were for 2021 or 2020. All countries except Iceland had data enabling the estimation of chronic HBV infection in key populations.

Our analysis confirms that chronic HBV infection prevalence across the EU/EEA remains substantial, with an estimated prevalence of 0.7%, representing 3.2 million persons living with chronic HBV infection. However, considering previous ECDC estimates, both chronic HBV prevalence and the number of persons living with chronic HBV infection have decreased compared with 2005–2015, when the prevalence was estimated at 1%, affecting 3.6 million persons in the EU/EEA, excluding the United Kingdom [22]. This trend has also been observed globally, with chronic HBV prevalence decreasing due to advances in preventive measures such as improved vaccination coverage and widespread implementation of safe injection practices [1].

The analysis highlights considerable variation in national prevalence estimates, with a 10-fold variation across countries. A prevalence of over 2% was observed in four countries (Bulgaria, Cyprus, Lithuania and Romania), while the lowest prevalence estimates were found in Slovakia, followed by Finland, Germany and Iceland. This geographical variation in prevalence, with the highest estimates in the south and east of the EU/EEA and the lowest estimates in the north and west, has been reported previously in several studies, as summarised in the systematic review by Hofstraat et al. [23].

The estimated chronic HBV prevalence in the studied populations varied both within and between countries. Our approach synthesised data on migrant populations from all countries, not just countries of high endemicity. Among migrants, prevalence differed by a factor of 13 across countries but remained below 2% in most countries. Prevalence among migrants was particularly high (10.5%) in Cyprus, likely reflecting a high prevalence among specific migrant populations such as refugees and migrants from countries of high (> 5%) endemicity [24]. While variations in chronic HBV estimates among PWID and MSM were observed between countries, there were no clear geographical patterns. Among the countries studied, Bulgaria and Romania had the highest national prevalence of chronic HBV infection, with similarly high findings for PWID and MSM populations.

The Bayes’ theorem calculations also showed similar variations across countries. The probability of being a migrant among those living with chronic HBV infection ranged from 1% in Romania to 97% in Liechtenstein. Except for Cyprus, all the countries where migrants accounted for over three quarters of persons living with chronic HBV infection were in the north-western parts of Europe. This finding aligns with a previous analysis estimating the prevalence of chronic HBV infection among first-generation migrants in the EU/EEA [24], which found that migrant populations accounted for 25% of HBV cases and, moreover, that cases among migrants from intermediate and high-endemicity countries were particularly prominent in north-western European countries. This pattern, however, is highly dependent on migration flows and therefore likely to be dynamic.

The analysis indicated that Estonia and Slovakia had the highest proportion of PWID living with chronic HBV infection, with this group accounting for around one fifth of all infections. While information on vaccination coverage among PWID is lacking, the high prevalence of chronic HBV infection among this group suggests that HBV vaccination coverage and harm reduction practices have not been optimal in these countries. Estonia reported outbreaks of HIV among PWID in the beginning of 2000 [25,26], which suggests there was an increased risk of exposure to blood borne infections and further highlights the need for greater efforts to improve HBV prevention measures. The analysis indicated that Hungary, Malta and Slovenia presented the highest proportion of MSM among persons living with chronic HBV infection. These countries also reported the highest proportion of MSM among recently diagnosed persons living with HIV in 2022 [27]. Various factors may underly these findings including stigma, insufficient provision of preventive measures and poor access to local services targeting these population groups.

This marked heterogeneity in the distribution of persons living with chronic HBV infection across population groups underscores the importance of tailored public health interventions. The predominance of persons living with chronic HBV infection among non-key populations in most EU/EEA countries confirms the importance of population-wide strategies for prevention, testing and treatment in these settings.

Importantly, the substantial contribution of migrants to national HBV prevalence in many countries highlights the need to integrate local demographic insights with epidemiological data to provide targeted access to diagnosis and treatment services at the local level [28]. It is important that any barriers to healthcare for migrant populations, such as language, cultural or cost issues, are addressed and that services are developed in close partnership with the local community. Furthermore, based on the results of a recent modelling analysis, a team of researchers has questioned the use of the 2% HBV prevalence in the country of birth as the threshold for screening migrant populations, as recommended by ECDC and WHO [4,29]. The researchers concluded that there is sufficient evidence to support screening and treatment of all migrant populations in the EU, not only those from countries with an endemicity above 2% [30].

Although MSM and PWID account for fewer persons living with chronic HBV infection at the national level compared with non-key and migrant populations, elevated HBV prevalence in these population groups reinforces the need for continued efforts to improve access to testing, vaccination and other preventive measures for these groups, such as condoms and sterile injection equipment to prevent onward transmission among MSM with multiple sexual partners and among active injectors [1,31]. With a large proportion of persons with chronic HBV infection known to be undiagnosed in the EU/EEA [32], efforts to scale up testing are critical. While disaggregated data on HBV testing among key populations are lacking, limiting a granular understanding of unmet needs around diagnosis, a recent report has highlighted that policies for HBV testing of key populations are lacking across EU/EEA countries, suggesting this unmet need may be considerable [33]. The results of our analysis provide valuable information to help tailor local testing strategies and better meet these needs.

Comparing our Workbook Method estimates with those from the Global Burden of Disease Study 2019, our estimates were higher (with no overlap of uncertainty intervals) in three countries, lower in five countries, and unavailable for one country [34]. These discrepancies likely reflect differences in data sources, methodology and modelling assumptions between the two approaches, particularly with respect to the treatment of sub-populations at higher risk.

In comparison with estimates from the Polaris Observatory, our estimates were higher (with no overlap of uncertainty intervals) in three countries and unavailable for comparison in five countries. No countries showed lower estimates relative to Polaris Observatory [11]. However, since the Polaris Observatory’s estimates were also used as input data in our model, it is difficult to disentangle the extent to which our results reflect the Workbook Method outputs vs the influence of Polaris Observatory input data. A probable explanation for our higher estimates is the inclusion of migrants in our model, as this group is more likely to present higher chronic HBV prevalence rates than non-migrants in most EU/EEA countries.

Other studies have used the Workbook Method to derive their national HBV and chronic hepatitis C infection prevalence, each with some differences in methodology. Vriend et al. were the first to adapt the original UNAIDS workbook to estimate hepatitis C prevalence in the Netherlands [35], followed by Koopsen et al. [9], who also estimated the chronic HBV prevalence. Alongside different population definitions, the Dutch study included female sex workers as a key population, differentiated the PWID and MSM groups based on their HIV status and excluded individuals under 15 years old. Koopsen et al. obtained HBV prevalence estimates for the Netherlands (estimated year: 2016) slightly lower than ours for 2022 (0.34%; LB–UB: 0.22–0.40% vs 0.4%; LB–UB: 0.3–0.5%) [9]. However, our study used Koopsen et al.'s estimates for non-key population chronic HBV prevalence and PWID prevalence, which limits a clear comparison between the two studies as the estimates may be more similar than they would be if different input data sources had been used. Kremer-Flach et al. [10] also used the Workbook Method to obtain HBV prevalence estimates for Germany in 2013 applying the RKI-adapted workbook. As the RKI-adapted workbook served as the starting point for our analysis, it aligns more closely with our study's methodology, using similar key populations but with differences in age stratification and group definitions (e.g. MSM who live with HIV instead of MSM irrespective of the HIV serostatus). Kremer-Flach et al. estimated national HBV prevalence for Germany in 2013 to be 0.4% (LB–UB: 0.3–0.4%) [10], which is similar to our 2022 estimate for Germany, with overlapping upper and lower bounds.

The sensitivity analysis results are illustrative and context-specific, reflecting the underlying data and structural characteristics of the country under study. Therefore, they are not intended to be extrapolated across countries, and their interpretation should be confined to the country tested, as national contextual differences may lead to substantially different outcomes. Nevertheless, certain findings from the sensitivity analysis may be informative for other countries and warrant further exploration, either in future adaptations of the Workbook Method or through complementary modelling efforts. For example, expanding the age categories used to capture the impact of vaccination programmes—tested using data from Poland—suggested that relying solely on the under 5 years of age group may lead to an overestimation of prevalence. Additionally, as noted by some NCPs, some of the sensitivity analysis results provided a more representative picture of their national situation and would be chosen over the default Workbook’s data sources and format.

Taken together, the overall results and sensitivity analysis results suggest that the Workbook Method is an approach that can be easily applied to provide estimates of chronic HBV prevalence that are comparable to other differing methodological approaches. Its structure allows adaptation to settings with varying levels of data availability and epidemiological profiles, including the selective inclusion or exclusion of key populations. Importantly, the Workbook Method can be implemented using standard spreadsheet software and does not require advanced statistical expertise, making it accessible to non-modellers and suitable for use in regions with limited analytical capacity. As such, it represents a standardised yet adaptable tool that could support regional HBV prevalence estimation beyond the EU/EEA, especially for countries that have not been able to estimate the prevalence burden through other methods.

The adapted Workbook Method offers several advantages. It accounts for varying chronic HBV prevalence among migrant populations, a factor often overlooked in national estimates derived from general population studies. These studies often underrepresent the contribution of migrant populations from intermediate- and high-endemicity regions to national chronic HBV prevalence in many European countries. By estimating chronic HBV prevalence in these groups based on country of birth, we could yield more precise estimates within this population, although the estimates of countries of birth from modelling studies may not be fully accurate and may not represent the chronic HBV prevalence of migrants in the EU/EEA country [9]. Importantly, including key populations in our model provided added value by highlighting the proportion of persons living with chronic HBV infection in different population groups within each country, which is critical information for public health action. Moreover, the Workbook Method does not require complex mathematical models or heavy data imputation, making it easily replicable by stakeholders with no or limited mathematical or statistical expertise. The workbook could, therefore, provide a simple approach for quickly reproducing estimates to monitor trends in chronic HBV prevalence over time and countries’ progress towards regional and global HBV elimination targets.

This method also has limitations. Primarily, it fails to address the overlap between population groups, potentially leading to an overestimation of chronic HBV prevalence at national level. Additionally, the accuracy of results is heavily contingent on the quality and availability of data sources and, specifically in this project, mostly rely on other modelled estimates rather than on up-to-date local empirical data that is likely to be more accurate. The limitations of the input data may necessitate further adaptation of the Workbook or the use of proxies to impute data for countries or populations with limited data availability. While more complicated modelling approaches also require robust input data, they can provide a more sophisticated approach to handling gaps in the data and can adapt data for changes over time from factors such as the impact of vaccination, treatment and mortality.

The adaptation of our Workbook was based on several assumptions that could considerably influence the final estimates. For example, we attempted to account for the effect of HBV vaccination on prevalence by stratifying the migrant and non-key populations into two age groups, using 5 years old as the cut-off. By doing so, we assumed that the chronic HBV estimates for the population aged 5 years or older already reflected the impact of vaccination among cohorts vaccinated in the neonatal period. However, the sensitivity analysis using the Polish adaptation, where the age cut-off closely matched the implementation of vaccination among newborns, suggested that the latter approach could result in lower and more accurate national chronic HBV estimates if relevant and up-to-date data were available. A further limitation of our approach is that we did not include persons in prison as a key population. While this population overlaps considerably with other key populations, such as PWID, persons in prison have distinct risks in relation to HBV infection. Future work could explore the inclusion of this population in a sensitivity analysis. Other assumptions and potential biases of our approach, including that migrants have the same chronic HBV prevalence as in their country of origin, the use of data from different years and the use of restricted age groups for MSM and PWID, are listed in Supplementary Table S2.

Further research employing more refined approaches such as compartmental models that consider infection states (susceptible, immunised, different infection statuses), age distribution and mortality rates across populations, risk of infection among different population groups and migration patterns should be considered. Refined models can provide more accurate estimates of chronic HBV prevalence and its distribution in the EU/EEA and within countries, inform HBV infection prevention and control policies and support the monitoring of progress towards HBV elimination targets. However, the availability of robust up-to-date empirical data for such models remains a challenge across the EU/EEA and consideration should be given towards addressing these data gaps.

## Conclusion

This study demonstrates the applicability of the Workbook Method for estimating chronic HBV prevalence across the EU/EEA, both at national level and among the key populations of migrants, MSM and PWID. The analysis confirms that the overall burden of chronic HBV infection remains considerable in the EU/EEA, with major differences between countries and key populations. These disparities likely reflect differences in the underlying epidemiology, as well as variation in the implementation of preventive measures and public health policies over time. The findings further underscore the complexity of the HBV situation in EU/EEA countries and the need for tailored, population-specific interventions.

While the results align with previous studies and are considered by countries to provide reasonable prevalence estimates, there remains a need for better empirical data and further research using refined models that overcome some limitations of our Workbook Method to improve accuracy. Accurate and up-to-date estimates of the prevalence of chronic HBV infection remain crucial for informing the effective scale up of prevention and control policies, analysing their impact and monitoring progress towards regional and global HBV elimination targets.

## Data Availability

All publicly available data used in this study are referenced in Supplementary Table S2. Additional data obtained directly from national contact points are available upon request.

## Supplementary Data

319000 Supplement
