# Supplementary material for: Use of the Workbook Method to estimate the prevalence of chronic hepatitis B infections in the European Union and European Economic Area, 2022
Source: Euro Surveill. 2026 Apr 9;31(14):2500322. doi: 10.2807/1560-7917.ES.2026.31.14.2500322 (PMC13074126; doi:10.2807/1560-7917.ES.2026.31.14.2500322)
Supplement: Supplement [file 25-00322_CANABARRO_Supplement.pdf]

Supplementary material

This supplementary material is hosted by *Eurosurveillance* as supporting information alongside the article **Use of the Workbook Method to estimate the prevalence of chronic hepatitis B infections in the European Union and European Economic Area, 2022**, on behalf of the authors, who remain responsible for the accuracy and appropriateness of the content. The same standards for ethics, copyright, attributions and permissions as for the article apply. Supplements are not edited by *Eurosurveillance* and the journal is not responsible for the maintenance of any links or email addresses provided therein.

Supplementary Table S1. Data sources used in the default workbook and the adaptations for each country.

**Default data sources**							
	**Population size**			**Hepatitis B prevalence**			
	**Total population**	**People who inject drugs**	**Men who have sex with men**	**Studied country**	**Other countries of origin (migrants)**	**People who inject drugs**	**Men who have sex with men**
**Default**	1) Eurostat (online data code: migr_pop3ctb) [1]: stratified by country of birth and age (<5 years old and all ages), in absolute values); 2022 (1st of January).	1) Thomadakis et al. [2], 2024: 15-79 years old; both sexes (in relative values; % of PWID in this age group); 2019.	1) Marcus et al., 2013 [3]: 15-64 years old; male (in relative values; % of MSM in this age and sex group); 2009	≤5-year-old group:<br>1) Polaris Observatory, 2023 [4] and country expert's feedback; 2022.<br>>5-year-old group:<br>1) Polaris Observatory, 2023 [4]: 2022.	All age groups:<br>1) Polaris Observatory, 2023 [4]: All countries presented in the study but Ukraine and United Kingdom, stratified by country of birth and age; 2022.<br>2) Trickey et al., 2023 [5]: Cyprus, Iceland, Latvia, Liechtenstein, Luxembourg; 2019.<br>3) ECDC personal communication, 2022: Ukraine; 2022.<br>4) UKHSA, 2023 [6]: United Kingdom; 2021.	1) EUDA, 2024 [7]: collection method and year vary among countries.	1) Trickey et al., 2023 [5]: modelled estimates; 2019.

Adaptations for each country							
**Country**	**Population size**			**Hepatitis B prevalence**			
	**Total population**	**People who inject drugs**	**Men who have sex with men**	**Studied country**	**Other countries of origin (migrants)**	**People who inject drugs**	**Men who have sex with men**
**Austria**	Default	Default	Default	Default	Default	Default. Routine diagnostic test; 2021.	Default
**Belgium**	Default	Default	Default	Default	Default	Default. Routine diagnostic test; 2014.	Default
**Bulgaria**	Default	Default	Default	Default	Default	Default. Seroprevalence; 2016.	Default
**Croatia**	1) Croatian Bureau of Statistics [8]: Population by country of citizenship; 2021.	Default	Default	Default	Default	1) Kolaric et al., 2010 [9]: seroprevalence; 2007.	Default
**Cyprus**	1) Cyprus Statistical Service [10]: stratified by country of birth (Cyprus x foreign country), 2022.	Default	Default	≤5-year-old group and >5-year-old group: Not applicable (no age stratification was used). General population: 1) Schweitzer et al., 2015 [11]: Modelled estimates; 2010. Additional calculations were conducted using information from the Unit for Surveillance and Control of Communicable Diseases (Cyprus Ministry of Health) regarding the migrant status of people living with chronic HBV infection between 2020-2022.	Default	Default. Routine diagnostic test; 2021.	Default
**Czechia**	Default	Default	Default	Default	Default	Default. Routine diagnostic test; 2021.	Default

Country	Population size			Hepatitis B prevalence			
	Total population	People who inject drugs	Men who have sex with men	Studied country	Other countries of origin (migrants)	People who inject drugs	Men who have sex with men
**Denmark**	1) Danmarks Statistik [12]: stratified by country of birth and age; 2022.	Default	Default. The estimate was considered to be a "questionable or unreliable value" in the study.	Default	Default	1) Trickey et al., 2023 [5]: modelled estimates; 2019.	Default
**Estonia**	Default; 2020	Default	1) Kasianczuk et a., 2023 [13]: Modelled estimates; 2021.	Default	Default	Default. Seroprevalence; 2017.	Default
**Finland**	Default	Default	Default. The estimate was considered to be a "questionable or unreliable value" in the study.	Default	Default	1) Trickey et al., 2023 [5]: modelled estimates; 2019.	Default
**France**	1) National Statistic Surveillance (dataset provided by in-country expert): stratified by country of birth and age; 2020.	Default	Default. The estimate was considered to be a "questionable or unreliable value" in the study.	1) Brouard et al., 2019 [14]: individuals born in France (mainland), 18-75 years old; 2016.	Default	1) UNODC, 2023 [15]: Seroprevalence; 2013.	Default
**Germany**	1) Federal Statistical Office of Germany, Population Census 2020 [16]: migrant populations stratified by country of birth; only individuals ≥18 years old; 2020.<br>2) Federal Statistical Office of Germany, Population Census, 2020 [17]: total population; 2020.	Default	Default; 2020	≤5-year-old group:<br>1) Default.<br>>5-year-old group:<br>1) Kremer-Flach et al., [date unknown] [18]: non-key adult population (≥18 years old); 2013; Workbook Method.	Default	Default. Seroprevalence studies; 2021.	Default

Country	Population size			Hepatitis B prevalence			
	Total population	People who inject drugs	Men who have sex with men	Studied country	Other countries of origin (migrants)	People who inject drugs	Men who have sex with men
**Greece**	1) Hellenic Statistical Authority, Population Census 2021 [19]: stratified by world regions based on the individual's citizenship; 2021; collected estimate	Default	Default	Default	Default	Default. Routine diagnostic test; 2022.	1) Communication with experts; HIV+/MSM, year not informed.
**Hungary**	Default	Default	Default	Default	Default	Default. Seroprevalence; 2015.	Default
**Iceland**	Default	Default	Not available	≤5-year-old group:<br>1) Imputed based on general population estimates and country's profile<br>>5-year-old group:<br>1) Trickey et al., 2023 [5]: modelled estimates; 2019.	Default	1) Trickey et al., 2023 [5]: modelled estimates; 2019.	Default
**Ireland**	1) Central Statistics Office Ireland, Census, 2022 [20]: stratified by country or continent of birth.	Default	1) Healthy Ireland Survey 2015 [21]; 2015.	1) Communication with experts; 2022.	Default	Current PWID:<br>1) Default. Seroprevalence; 2010.<br><br>Ex-PWID:<br>1) Long et al., 2016 [22]: Seroprevalence; 2001.	1) Casey et al., 2019 [23]: survey, 2017.
**Italy**	Default	Default	Default	Default	Default	1) UNODC, 2023 [15]: Seroprevalence; 2021	Default

Country	Population size			Hepatitis B prevalence			
	Total population	People who inject drugs	Men who have sex with men	Studied country	Other countries of origin (migrants)	People who inject drugs	Men who have sex with men
**Latvia**	Default	Default	Default	≤5-year-old group:<br>1) Imputed based on general population estimates and country's profile<br>≥5-year-old group:<br>1) Trickey et al., 2023 [5]: modelled estimates; 2019.	Default	Default. Seroprevalence; 2017.	Default
**Liechtenstein**	Default	1) Bruggmann et al., 2017 [24]: estimates of Switzerland; 2015.	Default. Estimates of Switzerland.	≤5-year-old group:<br>1) Electronic surveillance system of the Swiss Federal Office of Public Health: Estimates of Switzerland; 2023.<br>>5-year-old group:<br>1) Trickey et al., 2023 [5]: modelled estimates; 2019.	Default	1) Trickey et al., 2023 [5]: modelled estimates; 2019.	Default
**Lithuania**	Default	Default	Default	Default	Default	Default. Seroprevalence; 2018.	Default
**Luxembourg**	Default	Default	Default. The estimate was considered to be a "questionable or unreliable value" in the study.	≤5-year-old group:<br>1) Imputed based on general population estimates and country's profile<br>>5-year-old group:<br>1) Trickey et al., 2023 [5]: modelled estimates; 2019.	Default	1) Personal communication with in-country expert (February, 2024): collected estimates from four different harm reduction centres; 2019 [unpublished material].	Default

Country	Population size			Hepatitis B prevalence			
	Total population	People who inject drugs	Men who have sex with men	Studied country	Other countries of origin (migrants)	People who inject drugs	Men who have sex with men
**Malta**	1) Maltese National Statistics Office, Census 2021 [25]: stratified by country of birth and age (in absolute values); 2021.	Default	Default	Default	Default	1) UNODC, 2023 [15]: 2017.	Default
**Netherlands**	Default	Recent PWID:<br>1) Koopsen et al., 2019 [26]: Workbook Method; 2016.<br><br>Ex-PWID:<br>1) Default	Adam et al., 2024 [34]: Modelled estimates, 2022.	≤5-year-old group: Default.<br>>5-year-old group:<br>1) Koopsen et al., 2019 [26]: individuals at low risk of infection (≥15 years old); Workbook Method; 2016.	Default	1) Beuker at al., 2001 [27]: Seroprevalence; the Hague; 2000.	Default
**Norway**	Default	Default	Default. The estimate was considered to be a "questionable or unreliable value" in the study.	≤5-year-old group:<br>1) Personal communication and national registry; based on numbers of people living with chronic HBV infection among <5 years old children and vaccination coverage.<br>>5-year-old group:<br>1) Folkehelseinstituttet, 2023 [28]: estimates for Scandinavian population living in Oslo; 2001.	Default	Point estimate:<br>1) Folkehelseinstituttet, 2023 [28]: Seroprevalence; Oslo; 2023.<br><br>Uncertainties:<br>1) Folkehelseinstituttet, 2023 [28] and communication with expert: Seroprevalence; Oslo; 2015-2022.	Default

Country	Population size			Hepatitis B prevalence			
	Total population	People who inject drugs	Men who have sex with men	Studied country	Other countries of origin (migrants)	People who inject drugs	Men who have sex with men
**Poland**	1) Statistics Poland, Census 2021 [29]: stratified by country of birth; 2021.	Default	Default. The estimate was considered to be a "questionable or unreliable value" in the study.	≤5-year-old group: Default.<br>>5-year-old group:<br>1) Stepien, 2022 [30]: adult population; 2015.	Default	Default. Seroprevalence study; 2017.	Default
**Portugal**	1) Statistics Portugal, Census 2021 [31]: stratified by country or continent of birth and age; 2021.	Default	Default	Default	Default	Default. Routine diagnostic test; 2021.	Default
**Romania**	Default	Default	Default. The estimate was considered to be a "questionable or unreliable value" in the study.	Default	Default	Default. Seroprevalence; 2017.	Default
**Slovakia**	Default	Default	Default	Default	Default	Default. Seroprevalence; average of most recent years available (2019, 2020, 2021).	Default
**Slovenia**	Default	Default	Default. The estimate was considered to be a "questionable or unreliable value" in the study.	Default	Default	Trickey et al., 2023 [5]: modelled estimates; 2019.	Default

Country	Population size			Hepatitis B prevalence			
	Total population	People who inject drugs	Men who have sex with men	Studied country	Other countries of origin (migrants)	People who inject drugs	Men who have sex with men
**Spain**	Default	Default	Default	≤5-year-old group:<br>1) Ministerio de Sanidad, 2021 [32]: 2-5 years old; 2018.<br>>5-year-old group:<br>1) Ministerio de Sanidad, 2021 [32]: 20-80 years old; 2018.	Default	1) Observatorio Español de las Drogas y las Adicciones, 2023 [33]: seroprevalence; 2021.	Default
**Sweden**	Default; 2021.	Default	Default	≤5-year-old group:<br>1) Personal communication and national registry; on numbers of people living with chronic HBV infection among <5 years old children.<br>>5-year-old group:<br>1) Folkehelseinstituttet, 2023 [28]: estimates for Scandinavian population living in Oslo; 2001.	Default	Default. Routine diagnostic test; 2014.	Calculated as the mean of the modelled estimates of HBV prevalence among MSM in Denmark and Norway.

References for Table S1

1. European Commission E. Population on 1 January by age group, sex and country of birth (migr_pop3ctb). European Commission; 2024.
2. Thomadakis C, Gountas I, Duffell E, Gountas K, Bluemel B, Seyler T, et al. Prevalence of chronic HCV infection in EU/EEA countries in 2019 using multiparameter evidence synthesis. The Lancet Regional Health - Europe. 2024;36:100792.
3. Marcus U, Hickson F, Weatherburn P, Schmidt AJ, the EN. Estimating the size of the MSM populations for 38 European countries by calculating the survey-surveillance discrepancies (SSD) between self-reported new HIV diagnoses from the European MSM internet survey (EMIS) and surveillance-reported HIV diagnoses among MSM in 2009. BMC Public Health. 2013;13(1):919.
4. Razavi-Shearer D, Gamkrelidze I, Pan C, Jia J, Berg T, Gray R, et al. Global prevalence, cascade of care, and prophylaxis coverage of hepatitis B in 2022: a modelling study. The Lancet Gastroenterology & Hepatology. 2023;8(10):879-907.
5. Trickey A, Bivegete S, Duffell E, McNaughton AL, Nerlander L, Walker JG, et al. Estimating hepatitis B virus prevalence among key population groups for European Union and European Economic Area countries and the United Kingdom: a modelling study. BMC Infectious Diseases. 2023;23(1):457.
6. U. K. Health Security Agency. Hepatitis B in England – 2023 Report. Working to eliminate hepatitis B as a public health threat. London: UK Health Security Agency (UKHSA); 2023.

7. European Union Drugs Agency. Viral hepatitis elimination barometer among people who inject drugs in Europe. Lisbon: European Union Drugs Agency (EUDA); 2024.
8. Croatian Bureau of Statistics. Census of Population, Households and Dwellings in the Republic of Croatia, 2021. Zagreb, Croatia: Croatian Bureau of Statistics; 2021.
9. Kolarić B, Stajduhar D, Gajnik D, Rukavina T, Wiessing L. Seroprevalence of blood-borne infections and population sizes estimates in a population of injecting drug users in Croatia. Cent Eur J Public Health. 2010;18(2):104-9.
10. Statistical Service of Cyprus. Population Statistics. Nicosia, Cyprus: Statistical Service of Cyprus; 2025.
11. Schweitzer A, Horn J, Mikolajczyk RT, Krause G, Ott JJ. Estimations of worldwide prevalence of chronic hepatitis B virus infection: a systematic review of data published between 1965 and 2013. The Lancet. 2015;386(10003):1546-55.
12. Statistics Denmark. Population Statistics (FOLK1C). Copenhagen, Denmark: Statistics Denmark; 2025.
13. Kasianczuk M, Lõhmus L, Salekešin M, Rüütel K. Men who have sex with men population size estimation in Estonia. Tallinn: National Institute for Health Development; 2023.
14. Brouard C, Saboni L, Gautier A, Chevaliez S, Rahib D, Richard J-B, et al. HCV and HBV prevalence based on home blood self-sampling and screening history in the general population in 2016: contribution to the new French screening strategy. BMC Infectious Diseases. 2019;19(1):896.
15. United Nations Office on Drugs and Crime. World Drug Report 2023: Statistical Annex. UNODC; 2023.
16. Federal Statistical Office of Germany. Bevölkerung und Erwerbstätigkeit - Ergebnisse des Ausländerzentralregisters (Population and employment - results of the Central Register of Foreigners). Wiesbaden, Germany: Federal Statistical Office of Germany; 2021.
17. Federal Statistical Office of Germany. Daten aus dem Gemeindeverzeichnis - Kreisfreie Städte und Landkreise nach Fläche, Bevölkerung und Bevölkerungsdichte (Data from the municipal directory - independent cities and rural districts by area, population and population density). Wiesbaden, Germany: Federal Statistical Office of Germany; 2021.
18. Kremer-Flach K, Zimmermann R, An der Heiden M, Dudareva S. Estimated number of people infected with hepatitis B and C virus in Germany in 2013: a baseline prevalence estimate using the workbook method. Frontiers in Public Health. 2025;13:1471256.
19. Hellenic Statistical Authority. Official Statistics of Greece. Athens, Greece: Hellenic Statistical Authority; 2025.
20. Central Statistics Office Ireland. CSO Open Data Portal. Cork, Ireland: Central Statistics Office Ireland; 2025.
21. Department of Health. Healthy Ireland Survey 2015: Summary of Findings. Dublin: Government Publications; 2015.
22. Long J, Keenan E, Grogan L, Mullen L, Barry J, Sinclair H. HIV infection among heroin users and area of residence. Ir Med J. 2006;99(8):230-3.
23. Casey C, O'Donnell K, Brady M, Igoe D. EMIS-2017 Ireland: Findings from the European Men who have sex with men Internet Survey (Ireland). Dublin: Health Protection Surveillance Centre; 2019.
24. Bruggmann P, Blach S, Deltenre P, Fehr J, Kouyos R, Lavanchy D, et al. Hepatitis C virus dynamics among intravenous drug users suggest that an annual treatment uptake above 10% would eliminate the disease by 2030. Swiss Med Wkly. 2017;147:w14543.
25. National Statistics Office Malta. Official Statistics of Malta. Valletta, Malta: National Statistics Office Malta; 2025.
26. Koopsen J, van Steenbergen JE, Richardus JH, Prins M, Op de Coul ELM, Croes EA, et al. Chronic hepatitis B and C infections in the Netherlands: estimated prevalence in risk groups and the general population. Epidemiology and Infection. 2019;147:e147.
27. Beuker RJ, Berns MPH, Watzeels JCM, Hendriks V, de Coster EJM, Tonino-van der Marel E, et al. Surveillance van HIV-infectie onder injecterende druggebruikers in Nederland: meting Den Haag 2000. Bilthoven, Netherlands: Rijksinstituut voor Volksgezondheid en Milieu (RIVM); 2001.
28. Folkehelseinstituttet. Statusrapport om eliminasjon av hepatitt B og C som folkehelseproblem i Norge (Status report on the elimination of hepatitis B and C as a public health threat in Norway). Oslo: Folkehelseinstituttet; 2023.
29. Statistics Poland. Size and Demographic-Social Structure in the Light of the 2021 Census Results. Warsaw, Poland: Statistics Poland; 2021.

30. Stepien M, Zakrzewska K, Kolakowska-Kulesza A, Trzcinska A, Pancer K, Rosin M. Monitoring chronic hepatitis B prevalence through combination of seroprevalence and routine surveillance data: experience from Poland. 2022:202.
31. Statistics Portugal. Official Statistics of Portugal. Lisbon, Portugal: Statistics Portugal (INE); 2025.
32. Ministerio de Sanidad. 2º Estudio de Seroprevalencia en España. Madrid: Ministerio de Sanidad; 2021.
33. Observatorio Español de las Drogas y las Adicciones. Informe 2023. Alcohol, tabaco y drogas ilegales en España. Madrid: Ministerio de Sanidad. Delegación del Gobierno para el Plan Nacional sobre Drogas; 2023.
34. Adam PCG, Op de Coul ELM, Zantkuijl P, Xiridou M, Bos H, Blom C, Ketsuwan I, te Wierik MJM, David S, de Wit JBF. A survey-based assessment of rates and covariates of mpox diagnosis and vaccination provides evidence to refine eligibility criteria for mpox vaccination among gay, bisexual and other men who have sex with men in the Netherlands. Front Public Health. 2024;12:1194844. doi:10.3389/fpubh.2024.1194844

Supplementary Table S2. Assumptions and potential biases of the default workbook.

Population	Problem	Assumption	Reasons	Bias (direction and magnitude)	Sensitivity analyses results	Does it vary per analysed country?
**General**	Each data source collected or estimated the variable in/for different years.	Populations sizes and chronic HBV prevalence have not varied importantly over the years.	The estimation year is based on the migrant dataset, considering that migration patterns can significantly change each year. Additionally, due to the large size of the migrant population, it can significantly influence the estimates.<br>However, PWID and MSM population sizes, as well as all chronic HBV prevalence rates, have not varied significantly throughout the years.	Unpredictable direction and magnitude.	Not applicable	Yes
	Overlap between key populations.	There is no overlap between the key populations.	Most countries do not have data on key population's overlap.	Overestimation of the national prevalence. Unknown magnitude.	Not applicable	Yes. Countries with bigger key population sizes might have more individuals counted twice and overestimated national chronic HBV prevalence.
	Eurostat presents inconsistent values on ≥5 years old population.	All individuals in the general population that are not <5 years old (according to Eurostat), are ≥5 years old.	When summing up the populations <5 years old and ≥5 years old in Eurostat, the final count does not always equal to the total population size.	Overrepresentation of ≥5 years population and overestimation of people living with chronic HBV infection and, therefore, chronic HBV prevalence. Unknown magnitude.	Not applicable	Yes; not all countries presented this problem.
	Main data sources for migrant and non-key population have different age thresholds (Polaris Observatory uses ≤5 years as the age threshold, whereas Eurostat uses <5 years).	The chronic HBV prevalence of individuals aged 5 years old is the same of those aged <5 years old.	It is expected none or minimum difference in chronic HBV prevalence between the cohorts 5 years old and <5 years old.	Null or minimum	Not applicable	Yes. Not all the countries used these data sources.

Population	Problem	Assumption	Reasons	Bias (direction and magnitude)	Sensitivity analyses results	Does it vary per analysed country?
**General**	Effect of HBV vaccination is not addressed in the workbook.	Chronic HBV estimates for the population ≥5 years old already accounts for the impact of vaccination among the cohorts vaccinated against HBV in the neonatal period.	1) Default data source for chronic HBV prevalence (Polaris Observatory) does not provide information on chronic HBV prevalence disentangled by vaccination status or in different age groups.	Overestimation of chronic HBV prevalence among ≥5 years old.	[Tested country: Poland] Our assumption caused overestimation of national population estimate.	Yes. It might vary depending on the implementation of neonatal vaccination against HBV.
**Migrant**	No information about chronic HBV prevalence in country of birth.	Countries lacking chronic HBV prevalence data were assumed to have no one living with chronic HBV infection, as the corresponding cells were left unfilled.	a) The Workbook Method offers a straightforward way to calculate chronic HBV prevalence. Imputation complicates the calculation, diverging from our goal; b) Since the number of countries lacking information about chronic HBV infection is minimal, and likely corresponds to a low migrant population, our decision's impact on final estimates may be inconsequential.	Although this assumption might have resulted in lower estimates (given most of countries of birth have higher endemicity compared to the country analysed), the magnitude of the bias is potentially low.	Not applicable	Yes, in magnitude
	No information about migrant's country of birth and, therefore, their chronic HBV prevalence.	Migrants with no information about their country of birth have the same chronic HBV prevalence as the analysed country.	a) The number of migrants with no reported country of birth varies between the countries analysed. The more conservative and less elaborated approach is to use the analysed country's chronic HBV prevalence. For studied countries where only the continent of birth was informed, the median of the continent and ±40% for uncertainties were calculated based on the chronic HBV prevalence of countries belonging to the continent.	Unpredictable direction (it is dependent on the country analysed and which countries of birth were not represented); unpredictable magnitude.	Not applicable	Yes, in magnitude mostly
	Use of different data sources.	Differences in the year of estimation, method of estimation/collection, and biases of the different data sources are irrelevant for the final estimates.	There is not a single data source that provides all the information needed.	Unpredictable direction and magnitude.	Not applicable	No

Population	Problem	Assumption	Reasons	Bias (direction and magnitude)	Sensitivity analyses results	Does it vary per analysed country?
**Migrant**	No information on undocumented migrants.	Undocumented migrants were not accounted in our calculations or were accounted as "Unknown" or as local populations.	Information not available in the data sources used.	This assumption might have resulted in lower estimates, given that there might be a higher likelihood of undocumented migrants coming from countries with higher endemicity compared to the country analysed. Magnitude is potentially low.	Not applicable	Yes, in magnitude
	No information about chronic HBV prevalence in overseas territories.	Overseas territories have unknown chronic HBV prevalence and therefore should be listed under the "Unknown" country of birth in the migrant sheet.	a) There is a small representation of migrants born in overseas territories<br>b) This is the approach that requires less manipulation of data.	Potential underestimation of chronic HBV prevalence, but of minimal or null magnitude.	[Tested country: Malta] Our assumption did not cause any impact on the chronic HBV estimates.	Unknown
	Some countries might have incomplete inaccurate data on Schengen migrants (EU/EEA citizens).	If the analysed country does not report Schengen migrants, they are not classified by country of birth and are reported as "Unknown".	Information might be incomplete in the data sources used.	Unpredictable direction and magnitude.	Not applicable	Unknown
	Population size and HBV prevalence were obtained from a single reference year and does not capture temporal changes in migration patterns or changes in HBV prevalence in countries of origin.	The migrant population size and HBV prevalence in countries of origin remain relatively stable over the years and are represented in the single-point estimates.	The dynamic updating of migrant population size and HBV prevalence was beyond the scope of the current study.	Unpredictable magnitude and direction.	Not applicable	Yes

Population	Problem	Assumption	Reasons	Bias (direction and magnitude)	Sensitivity analyses results	Does it vary per analysed country?
**Migrant**	No information on the chronic HBV prevalence of migrants living in the studied country. Additionally, we are disregarding any effect that migrating might cause to the risk of contracting HBV in the destination country (e.g. healthy migrant effect).	Migrant prevalence from country A is the same when coming to country X or to country Y. Migrant groups have the same chronic HBV prevalence of their country of birth; there is not a selection of healthier individuals. It is also possible that migrant groups may have a higher chronic HBV prevalence than their country of birth if for example there are limited prevention services provided for certain population groups such as MSM or PWID.	There is no information on the chronic HBV prevalence specifically on migrants in each country of destination.	Estimations among migrants might be overestimated; unpredictable magnitude.	[Tested country: Germany] Our assumption caused underestimation of migrant and national population estimates.	Yes
**PWID**	No information on chronic HBV prevalence among ex-PWID.	Recent and ex-PWID present the same chronic HBV prevalence.	a) There is no information on chronic HBV prevalence among ex-PWID;<br>b) HBV is a chronic infection and currently has no cure;<br>c) Vaccination might have not substantially impacted the age groups of PWIDs yet.	Null or minimum	Not applicable	Unlikely
	PWID population includes only individuals between 15-79 years old.	There are no PWID younger than 15 years old or older than 79 years old.	Data source used to estimate the prevalence of PWID used the age group 15-79-year-old	Estimation of the PWID population might be too low; magnitude is low	Not applicable	Unlikely
	Chronic HBV prevalence was estimated using different methods in each country.	The methods used by each country to estimate the chronic HBV prevalence among PWID do not importantly affect its estimates.	The countries have not followed a standard approach/method to estimate chronic HBV prevalence in this population.	Unpredictable direction and magnitude.<br>Hinders comparison between countries.	Not applicable	Not applicable

Population	Problem	Assumption	Reasons	Bias (direction and magnitude)	Sensitivity analyses results	Does it vary per analysed country?
**MSM**	MSM population includes only individuals between 15-64 years old.	There are no MSM younger than 15 years old or older than 64 years old.	Data source used to estimate the prevalence of MSM used the age group 15-64-year-old.	Estimation of the MSM population might be too low (especially among older MSM); magnitude is potentially low.	Not applicable	Unlikely
	Individuals do not report or identify themselves as MSM.	MSM population size was accurately measured, and models have addressed the risk of underreporting.	Stigma towards MSM might result on MSM underreporting.<br>Individuals do not identify themselves as MSM and do not report belonging to this group.	Estimation of the MSM population might be too low; magnitude is unknown.	Not applicable	Potentially. It might be higher in countries where stigma and discrimination towards MSM are more prevalent.
	The data source used for MSM population size estimates is old (2009).	The MSM population size across EU/EEA countries has remained the same since 2009.	There are no more recent and comparable estimates are not available for most countries. Additionally, national experts were unable to suggest alternative data sources for MSM population size estimates.	Unpredictable direction and magnitude.	Not applicable	Likely

Supplementary Table S3. Comments received from the hepatitis B National Contact Points regarding their workbook chronic HBV estimates.

Country	Comment
**Austria**	No additional comments.
**Belgium**	HBV prevalence among PWID: The value used was obtained from a small sample size in a single treatment centre in Antwerp, which might not be representative of the country. The HBV prevalence in the non-key population in Belgium is likely to be lower than the 0.5% estimate used**,** as a lower value was estimated in a soon to be published study, which found high levels of HBsAg false positivity.
**Bulgaria**	No additional comments.
**Croatia**	No additional comments.
**Cyprus**	There is intense influx and outflux of migrants in the country (approximately 2,000 per month), which can affect the HBV prevalence in this group and nationally.
**Czechia**	HBV prevalence among non-migrants is likely to be overestimated due to the failure to include the impact of vaccination and the significant disappearance of the oldest age cohorts in which the prevalence of chronic hepatitis B has historically been highest.
**Denmark**	According to our capture-recapture study the Danish estimate is 0.3%, but this does not include persons <15 years of age. Also, the estimate dates back to 2016 (PMID: 36221255)
**Estonia**	No additional comments.
**Finland**	The results appear somewhat unexpected; however, it is not possible to fully assess them at this time.
**France**	National HBV prevalence is overestimated, potentially because of overlap of key populations.
**Germany**	The data seem plausible and we agree with these calculations.
**Greece**	No additional comments.
**Hungary**	No additional comments.
**Iceland**	No additional comments.
**Ireland**	The overall national estimate for Ireland looks realistic, but some of the individual country of birth breakdowns are not likely to be accurate and the degree of certainty around the PWID and MSM sub-population estimates is lower.
**Italy**	No additional comments.
**Latvia**	No additional comments.
**Liechtenstein**	In Liechtenstein there is only a rural area. The biggest community regarding the size of the population consists of 6.000 inhabitants. Therefore, certain limitations must be considered when extrapolating the Swiss numbers.
**Lithuania**	No additional comments.

Country	Comment
**Luxembourg**	We observed influx and outflux of migrants every year, coming from different countries that could change every year. This could affect the prevalence in this population.
**Malta**	No additional comments.
**Netherlands**	No additional comments.
**Norway**	The prevalence among 'non-key populations' comes from a study in Oslo and may not be nationally representative. In addition, this prevalence estimate may be overestimated, as it is based on prevalence data from 2001, after which time a range of preventative measures have been introduced, including routine childhood immunisation.<br>The prevalence data among PWID (2023) comes from a study in Oslo and may not be representative of all PWID nationally.
**Poland**	National HBV prevalence is overestimated, potentially because of the methodology that did not consider the impact of vaccination coverage on HBV prevalence. Since the Polish population has been relatively homogeneous for over 20 years of HBV vaccination of infants, and the impact of migration from countries with a high prevalence of HBV was of low level, the scenario taking into account the impact of vaccination coverage on the national HBV prevalence seems more accurate.
**Portugal**	It is possible that the national prevalence of HBV is overestimated due to the overlap of key populations.
**Romania**	No additional comments.
**Slovakia**	No additional comments.
**Slovenia**	No additional comments.
**Spain**	Estimates could be overestimated due to the methodology not considered the impact of vaccination (see reference: Domínguez A et al. Impact of the Universal Implementation of Adolescent Hepatitis B Vaccination in Spain. Vaccines 2024, 12, 488. https://doi.org/10.3390/vaccines12050488)
**Sweden**	There is an important overlap of MSM, PWID and migrant populations in the country.<br>The methodology does not consider the impact of vaccination and other preventive measures.<br>Underestimation of HBV prevalence among MSM.<br>Regarding the HBV prevalence among PWID, there is a risk of overestimation, as it is based on regional prevalence data from before a range of preventive measures were implemented. Additionally, the regional data may not be representative at the national level.
